# Clinical, Cardiological and Serologic Follow-Up of Chagas Disease in Children and Adolescents from the Amazon Region, Brazil: Longitudinal Study

**DOI:** 10.3390/tropicalmed5030139

**Published:** 2020-08-31

**Authors:** Ana Yecê das Neves Pinto, Vera da Costa Valente, Sebastião Aldo da Silva Valente, Tamires Anastácia Rodrigues Motta, Ana Maria Revorêdo da Silva Ventura

**Affiliations:** 1 Instituto Evandro Chagas/Secretaria de Vigilância em Saúde/Ministério da Saúde-Brasil, Ananindeua 67030-000, Pará, Brazil; veravalente@iec.gov.br (V.d.C.V.); aldovalente@iec.gov.br (S.A.d.S.V.); ana_mariaventura@hotmail.com (A.M.R.d.S.V.); 2 Departamento de Saúde Integrada, Universidade do Estado do Pará, Belém 66050-540, Pará, Brazil; anastasia.motta@gmail.com

**Keywords:** Chagas disease, Cohort studies, Neglected diseases, *Trypanosoma cruzi*, Dynamic programming

## Abstract

**Background:** Outbreaks of Chagas disease (CD) by foodborne transmission is a problem related to deforestation, exposing people to triatomines infected by T. cruzi, in the Amazon region. Once involving long-time follow-up, the treatment efficacy of the CD during its acute phase is still unknown. The authors aim to describe the clinical and epidemiologic profile of children and adolescents with CD, as well as treatment and cardiac involvement during the follow-up. **Methods**: A descriptive cohort study was conducted from 1998 to 2013 among children and adolescents up to 18 years-old with confirmed diagnosis of CD. All participants met the criteria of CD in the acute phase. **Results**: A total of 126 outpatients were included and received treatment and follow-up examinations during a medium period of 10.9 years/person. Most of them (68.3%) had their diagnosis established during oral transmission outbreaks. The diagnostic method with the most positive results rate (80.9%) was the IgM class anti-*T. cruzi* antibody test as an acute phase marker, followed by the thick blood smears (60.8%). Acute myopericarditis was demonstrated in 18.2% of the patients, most of them with favorable evolution, though 2.4% (3/126) persisted with cardiac injury observed at the end point of the follow-up. **Conclusions**: Antibodies against *T. cruzi* persisted in 54.8% of sera from the patients without prognostic correlation with cardiac involvement. Precocious treatment can decrease potential cardiac complications and assure good treatment response, especially for inhabitants living in areas with difficult accessibility.

## 1. Introduction

Since 1996, there has been an increase in the number of cases of Chagas disease (CD) in Brazil, especially in the states Pará and Amapá in the Brazilian Amazon. There, CD has shown different transmission patterns from other regions, with predominant presentation in focal recurrent and seasonal episodes affecting persons of the same family or neighbors, mainly by contaminated food. This emerging route of transmission has been the subject of innumerous epidemiological studies that added, over ten years, sufficient epidemiological evidence to explain some of these episodes as a consequence of accidental food contamination involving fruits ingested in natura under poor hygienic conditions [1].

In the past, data from most of the endemic areas of Chagas disease in Brazil recorded the acute phase of the disease as an inapparent form among children, on contrary of those cases occurred in the Amazon region [2,3]. In Pará state, the disease has peculiarities that are expressed as a very differentiated clinical entity of chronic disease with high morbidity. It is characterized by acute febrile syndrome, with manifestations of fever, chills, headache and myalgia and subcutaneous edema, in addition to reversible acute cardiac commitment, after treatment [4]. Chronic phase-type records of dilated cardiomyopathy are rare in the Amazon region [3].

For epidemiological surveillance, the precocious access to diagnosis is an important strategy regarding the precocious treatment. Nonspecific clinical features may be one of the reasons for low suspicion and late diagnosis [5,6,7]. This delay also is due to the low capacity of regional health professionals in including the patients into the diagnosis flows. In addition, since CD is inserted in a historical context of chronic illness, frequently the physician mis suspicion the febrile acute phase of the disease.

The description of CD related to all age groups will help the health professionals to increase knowledge about its morbidity profile, including cardiac complications, especially in the case of suspicious diagnosis, which will make a precocious diagnosis possible. In addition, the clinical descriptions of endemic areas of classical vector CD transmission no longer satisfy the unusual Amazonian epidemiology of this disease, requiring that the new descriptions be widely disseminated. In this sense, the objective is to characterize a historical cohort of children and adolescents under 18 years of age regarding their follow-up related to immediate and mediate treatment, until the outcome. The authors hope that a detailed description of the clinical profile of children and adolescents with CD here reported may be an useful tool as an evidence to strengthen the trainings of professionals in the management/diagnosis of suspected cases and thus, contribute to minimize the delay in diagnosis of this age group.

## 2. Materials and Methods

Study population: It consisted in a prospective historical cohort study of patients with acute CD phase, diagnosed since 1996. All participants were treated and followed up in the reference service for the follow-up of CD patients in the state of Pará, which operates according to the Clinical Protocols on Chagas Disease (PCDCha) and is located at the Unified Medical Care Service–Instituto Evandro Chagas (SOAMU/IEC). All patients included in the study were enrolled according to the ethical precepts of human research on a voluntary basis in accordance with their legal representatives. The project was approved by the research ethics committee of the IEC (ethical approval code n. 655.002) in accordance with the CD clinical protocol.

Procedures: All the participants met the criteria of acute phase: persons confirmed with clinical (signs and symptoms of febrile acute disease), epidemiological (another member of family or neighbor confirmed with CD) and with direct or indirect positive parasitological tests and/or a serologic reagent test with an acute phase marker (IgM against *T. cruzi*).

Data collection was carried out by analysis of medical records of the individuals eligible for the study. The following demographic data were evaluated: origin of the infection; likely transmission; contact with insect vectors; maternal prenatal history in children under two years of age; food history in children under two years of age; previous family history of Chagas disease; acute phase clinical data with information on the onset and interval time of the disease, initial disease suspicion, predominant signs and symptoms, coinfections, previous diseases, diagnostic results, nonspecific tests results and acute myocardial damage with emphasis on electrical conduction disorders.

Three probable modes of transmission were considered by the authors: Oral (or vectorial-oral) transmission—occurred during an outbreak of CD related to accidental ingestion of triatomines feces or even smashed triatomine in beverages. In these outbreaks the children and their family members had their diagnosis simultaneously made); vectorial transmission—children with history of contact with triatomine vectors; vertical transmission—children up to one years-old whose mother was also infected.

**Figure 1:**
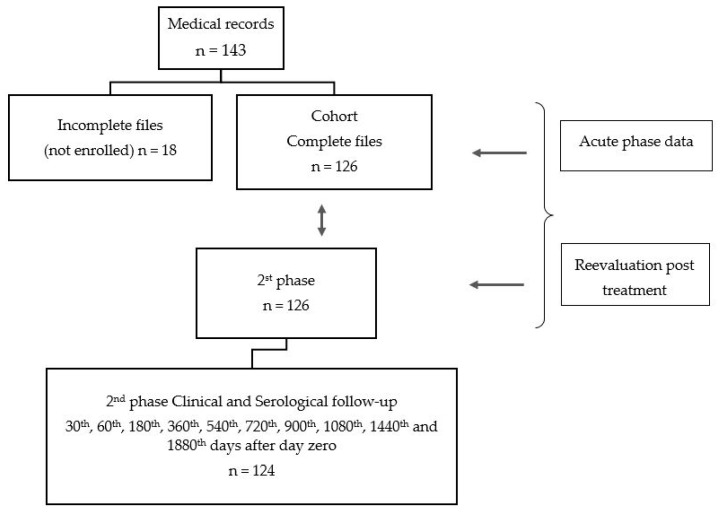
Study design.

Two phases composed the temporal cohort data: one representing all eligible outpatients with acute phase of CD and a second including the same patients in a phase called follow-up after treatment corresponding to a medium period 10.5 years per person. (See Figure 1—design)

The outcomes considered the following variables analyzed simultaneously: negative serologic conversion or persistence of IgG antibodies against *T. cruzi* with or without disease; persistence of IgG antibodies against *T. cruzi* with heart disease compatible changes in electrocardiogram or in echocardiogram compatible with CD (See Methods).

Laboratory evaluation: The patients underwent a blood count, parasitological (quantitative buffy coat (QBC®) or thick blood smear) and serologic tests, performed at the time of diagnosis or shortly before starting treatment and repeated sequentially after treatment, including thick blood smear, blood culture and serologic tests for the detection of anti-*T. cruzi* IgM and IgG antibodies.

Serologic techniques were carried out using the indirect hemagglutination assay (IHA) kit Hemacruzi, Biomérieux (qualitative assay) and the indirect immunofluorescence assay (IIF)-kit Imunocruzi, Biomérieux for titration (quantitative assay) of IgM and IgG classes of immunoglobulins. For the latter, anti-human IgM and IgG labeled with fluorescein (BIOLAB, Brazil) were used. For IHA, a titer of 1:40 was tested and for IIF, sera dilutions from 1:40 up to 1:1280 were tested. The reference value for both tests was a nonreagent result at the 1:40 or less dilution.

The serologic follow-up post treatment was analyzed cumulatively according to individual evaluations. These were performed according to service protocols on the first day of treatment (day zero) and sequentially on the 30th, 60th, 180th, 360th, 540th, 720th, 900th, 1080th, 1440th and 1880th day after day zero.

The evaluation also included blood analysis and platelet measures. Hemoglobin rate according to age and gender determined anemia (Hb < 11 g% in children from 6 months up 9 years of age; <12 g% in female adolescents; <13 g% in male adolescents).

The normal reference values to the leucocyte counts ranged from 5000 to 10,000 /mm^3^. Values below or above this range were defined as leukopenia and leukocytosis. The normal reference values for platelet levels ranged from 150,000 to 300,000 /mm^3^. The values below or above this range defined thrombocytopenia or thrombocytosis.

For the clinical classifications of cardiac involvement levels during the acute phase, only the results of electrocardiograms (EKG) and echocardiograms from this phase were evaluated. The echocardiograms were used only to classify the acute stage, but not for the follow-up. To this analysis, the parameters already described in Amazonian populations [5] were considered:

(1) Severe cardiac involvement: heart failure caused by chagasic infection as evidenced by echocardiogram results demonstrating severe myopericarditis with pericardial effusion;
(2) Moderate cardiac involvement: myocarditis accompanied by sinus tachycardia or other arrhythmias with pericarditis and pericardial effusion;
(3) Mild cardiac involvement: myocarditis accompanied by sinus tachycardia or other simple conduction disorders.

The outcomes were classified by the analysis of three parameters: (a) negative serologic conversion, for those with a sequence of three negative serologic antibodies test in two different methods; (b) undefined, for those with loss of serologic follow-up; (c) persistence of IgG antibodies against *T. cruzi* without evidence of disease; (d) persistence of IgG antibodies against *T. cruzi* with cardiac disease compatible with CD.

Analysis: To evaluate the serologic follow-up time, the mean follow-up time per person-year was calculated, considering the year of inclusion into the study, subtracted from the most recent evaluation year (2013) and divided by the number of years elapsed between primary inclusion (1998) through the most recent evaluation (e.g., 2013). Thus, from these calculations, the mean follow-up time per year-person after treatment was 10.9 years.

The geometric means of anti-*T. cruzi* IgG antibody titers as measured by IIF were compared on two different points of time, based on day zero as the start of treatment. Therefore, the measurements were compared on days zero and 720th or two years after treatment and on days zero and 1800th or five years after treatment, using analysis of variance (ANOVA) test, using 5% as the significance level.

## 3. Results

### 3.1. Demographic Data, Origin and Spatial Distribution

**Figure 2:**
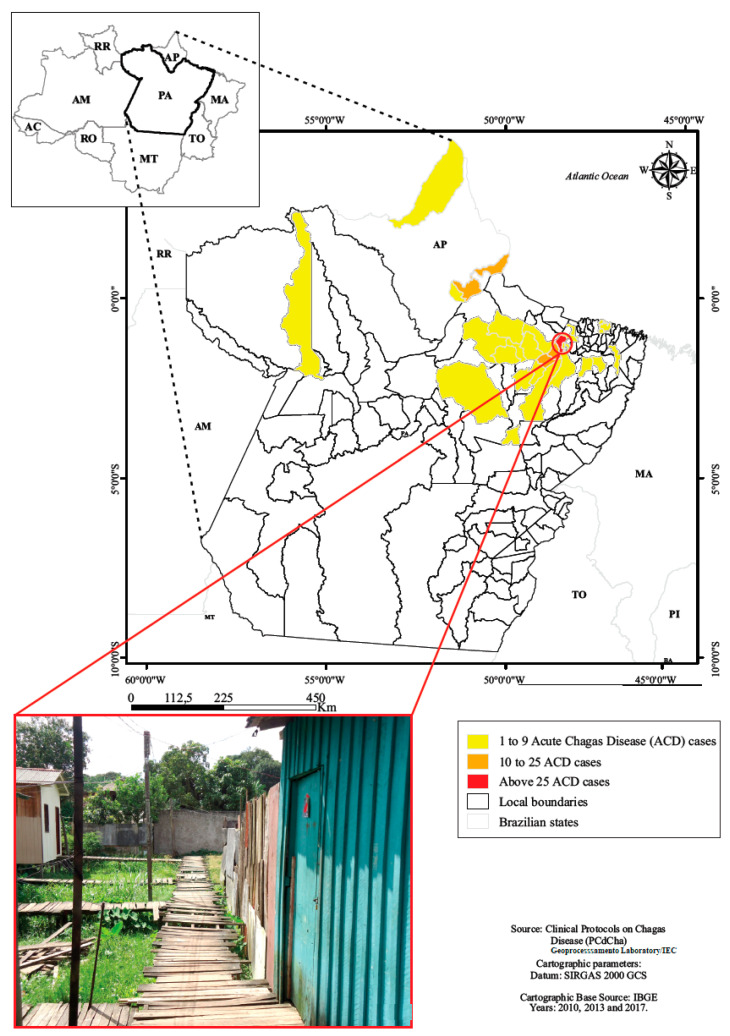
Residence location of children and adolescents with acute Chagas disease, Belém, Pará, Amazon region, Brazil.

A total of 126 children and adolescents from 0 up to 18 years of age treated during the acute phase of the disease were evaluated from 1998 to 2013.The origin of infection were located in both urban and rural municipal areas of Pará and one city of Amapá state (Table 1, Figure 2).

The mean follow-up time per person-year after treatment was 10.9 years. The mean period of illness from the onset of symptoms to the diagnosis was 33.2 days.

### 3.2. Clinical Features

The most frequent clinical manifestations among the participants were acute febrile syndrome in 92.8% and cardiac involvement in 16.1% (Table 2).

**Figure 3:**
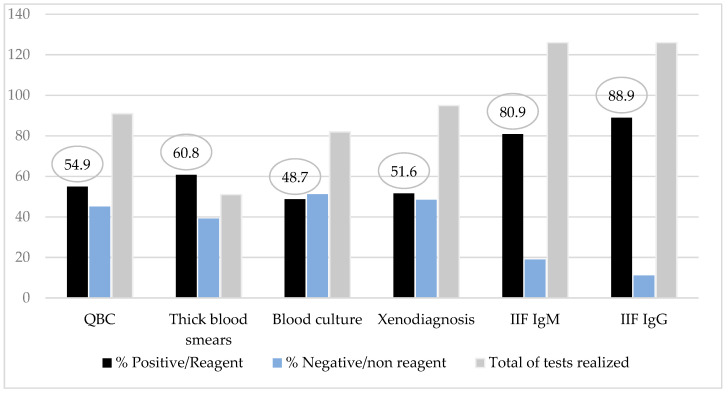
Diagnostic methods results rate from children and adolescents with Chagas disease.

Direct parasitological examinations revealed a positivity rate of 60.8% by the thick blood smears and 54.9% by the QBC® method. The method with most high positivity rate was the IgM class of anti-*T. cruzi* antibodies test as an acute phase marker (80.9%) (Figure 3). Among patients with IgM tests nonreagent (24/126), all of them had parasitological test positive/reagent and/or serologic test IgG positive added to a clinical signs of the disease.

Anemia with hemoglobin values of 7.6 mg/dL was the most frequent finding among hematological abnormalities (Table 3).

### 3.3. Acute Phase Cardiac Involvement

Among 88 patients who performed a complete evaluation with at least one of the two cardiac exams, i.e., EKG or echocardiogram, 18.2% (16/88) revealed some cardiac involvement and were classified according to clinical and electro- or echocardiographic results.

In 52.9% of the patients, the resting electrocardiogram showed a normal result during the acute phase. Among those with altered exams, there was a higher frequency of diffuse repolarization and sinusal tachycardia (Table 4). On those submitted to echocardiogram, we observed 18.9% (16/88) with pericardial effusion, of whom 75.0% (12/16) had severe, 18.8% (3/16) mild and 6.2% (1/16) moderate cardiac involvement.

### 3.4. Response to Treatment, Adverse Effects and Serologic Follow-Up after Treatment

**Figure 4:**
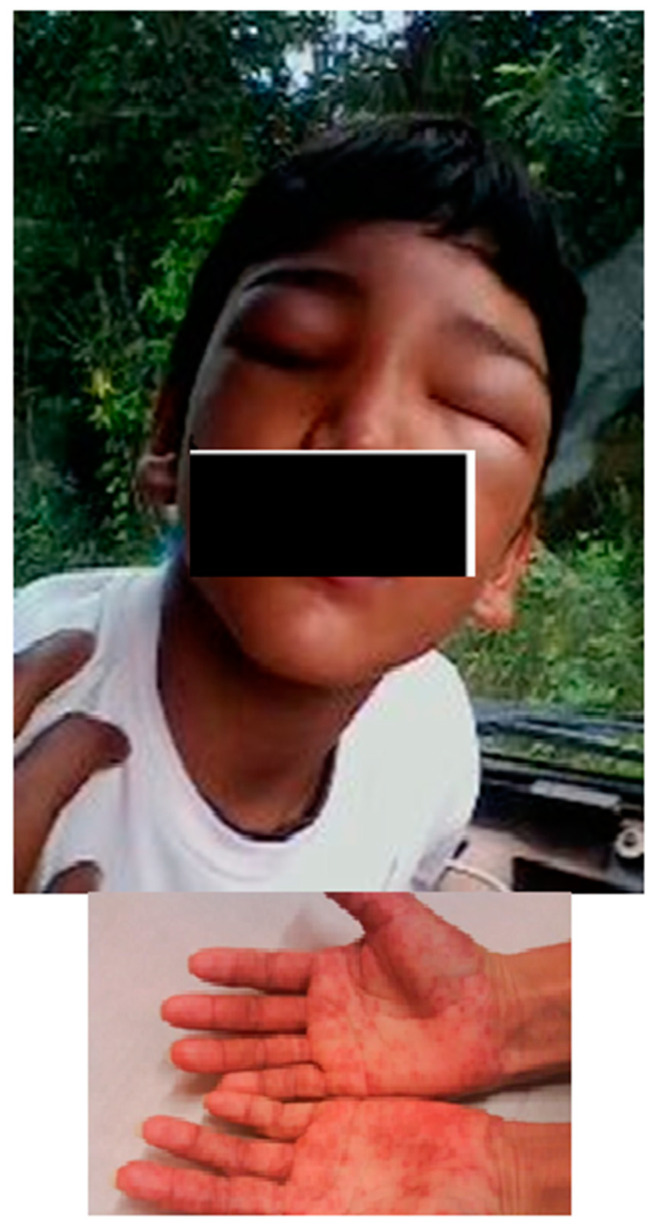
Face angioedema and maculopapular rush in hands post treatment with benznidazole, in two patients.

Once the children were diagnosed, they were immediately treated within a medium period of 20.8 h, with a minimum of 2 h and maximum of 72 h. A total of 123 patients (97.6%) were treated with benznidazole and one was treated with nifurtimox, due to severe benznidazole adverse effect (described as follow). The study revealed that 94.3% of the patients (117/124) received treatment with drugs during 50 or more days, while only 5.6% (7/124) for less than 49 days. Among those who were treated for less than 49 days, five of them did so for at least 30 days and two just for one day. Those two children that have incomplete treatment were disregarded in this analysis. Adverse reactions occurred in 20.2% (25/124) of the patients consisting of dermatologic alterations (72%); hair loss (3%); gastric disturbances and insomnia (2%) as principal findings. Dermatological alterations included: maculopapular rash, urticaria, morbilliform exanthema and angioneurotic edema (Figure 4).

## 4. Clinical Follow-Up According to Serologic and Cardiac Evaluation

**Figure 5:**
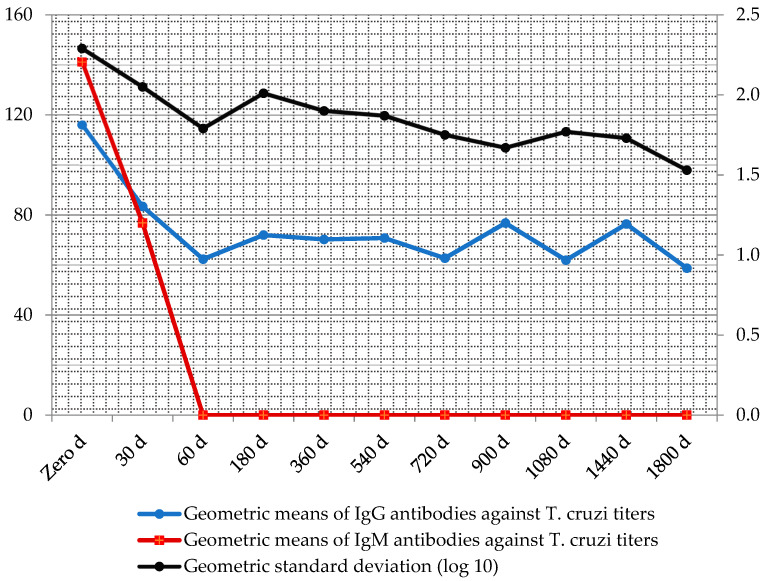
Follow-up geometric means of IgM and IgG antibody titers against *T. cruzi,* after treatment with antichagasic drugs measured by immunofluorescence absorbent tests.

Throughout the serologic follow-up, the geometric means of antibody titers after treatment declined in medians of up to three titles (Figure 5).

According to the outcome evaluations which included cardiac exams and serologic tests before and after treatment until the end point, 54.8% of the patients demonstrated sustained reactive IgG antibody titers (low titers) without evidence of cardiac disease after the mean period of 10.9 person–years of follow-up (Table 5). See definitions in Methods.

## 5. Discussion

In this observational study of monitoring patients over a period of 15 years, the conglomeration of patients in family outbreaks suggested oral transmission of 71.42%. In Brazil, there were 112 outbreaks in conglomerates between 2005 and 2013, with ingestion of contaminated foods as the most frequent form of transmission. However, more than 20% of the patients had no transmission information form registered, with 87.5% of them in the Pará state, due to failures of timely outbreak investigations [8].

Despite the smallest proportion of vector transmission (6.4%) in Brazil, persistence of focal points was observed, and entomological surveillance has been strengthened annually [8]. The national seroprevalence survey for the evaluation of CD control in Brazil conducted between 2001 and 2008 reported an infection prevalence of 0.03% among children under five years of age, in the majority suggesting vertical transmission due to maternal positive results and in the others indicating possible vectorial transmission [9,10]. In our sample, vector transmission was the second most frequent mode, as 71.4% of children and adolescents were infected during outbreaks with evidence of food transmission.

There were predominant manifestations of prolonged febrile syndrome, including the main triad of fever (92.85%), headache (58.73%) and cutaneous pallor (50.79%). Rassi & Ferreira (1971) reported fever as the main general manifestation, followed by ganglionic hypertrophy and subcutaneous edema in the same proportion of 45.94% in all their patients with a mean age of 12.1 years [11]. Pinto et al. (2007) corroborated that febrile syndrome is the principal clinical feature in 95% of patients with severe ACD, followed by dyspnea (75%) and asthenia (65%), in addition to other signs and symptoms, although the mean age of these patients was 46.7 years [12]. Shikanai-Yasuda et al. (1990) also registered a high frequency of fever (91.7%), lymph node enlargement (70.8%), hepatomegaly (66.7%), splenomegaly (41.7%), lower limb edema or generalized edema (62.5%), cutaneous rash (12.5%), cough (8.3%) and signs of congestive heart failure (16.7%) [13].

In patients previously studied in the Amazon region, the search for IgM and IgG antibody titers was essential for diagnosis in patients with prolonged disease because in these cases, the individual diagnosis was delayed, reducing the chance of parasite detection in the peripheral blood (5). In the series studied, the most effective tests to detect the infection were the serologic methods IgM and IgG against *T. cruzi* measured by IIF (80.9% and 88.9%), followed by parasitological methods thick blood smears (60.8%) and QBC® method (54.9%). This result strengths the recommended strategies of the Brazilian Chagas consensus about simultaneous serologic and parasitological procedures in suspected cases.

Anemia occurred in almost half (49.2%) of the patients, with a minimum value of hemoglobin concentration of 7.0 g% and a maximum of 15.0 g%, with a mean of 10.7 g%, which is below the reference value. Leukometry was on reference limits and therefore more frequently found than the registers from another outbreak (37.5%) of ACD in urban areas described by Shikanai-Yasuda et al. (1990) [13]. Thrombocytosis was frequently found, both in patients from this sample as from other series [5].

Among those who underwent EKG examination during the acute phase 59.1% did not have electrocardiographic abnormalities. However, among those with alterations, the main findings were diffuse repolarization abnormalities in 27.8% of patients, followed by sinus tachycardia (16.7%). Comparatively, in the study by Noya et al. (2010) that included 77 children during an acute phase outbreak in Venezuelan schoolchildren, the incidence of alterations was 69.7%. The abnormalities found in 40.9% were lower than those reported by Noya et al. (2010) in an outbreak of ACD [14].

In our study, we found diffuse repolarization abnormalities (27.8%) and sinus tachycardia (16.7%), which is also different from the study by Noya et al. (2010), whose main changes were T and P wave segments (37.8%) and arrhythmias (32%). Additionally, in our series, sinus tachycardia proved to be a signal during physical examination as suggestive of suspected ACD.

Another study made in the Amazon region including adults and children observed 52.3% of electrocardiographic changes, which was three times higher than the frequency in the children of this sample. Comparatively, the main electrocardiographic findings among adults were diffuse ventricular repolarization abnormalities (DVRA) (43.3%) and low voltage of the QRS axis (15.6%), while in the children studied, the findings were DVRA and sinus tachycardia [15].

Negative serum conversion is a marker used in follow-up studies after treatment. Although controversial, anti-*T. cruzi* IgG antibodies persistence at low levels in serial evaluations is used as a marker of cure. In Argentina, for example, 90% of children who completed treatment in 60 days had a persistent decrease or disappearance of specific anti-*T. cruzi* antibodies [16]. Corroborating the Argentine study, the Brazilian series studied showed an excellent response to treatment considering the established clinical cure criteria, especially the complete resolution of the symptoms and the persistent decrease in titration of IgG antibodies against *T. cruzi*. Negative seroconversion was observed at a lower frequency. When compared to the Argentine study, the authors questioned negative seroconversion as a potential cure marker and propose the PCR methodology in view to improve the cure rates most faithful [17].

Currently, only two drugs have demonstrated efficacy for the treatment of CD: nifurtimox, a nitrofuran derivative and benznidazole, a nitroimidazole derivative. Benznidazole is well tolerated among children, being less susceptible to adverse effects than adults and tolerating higher doses [18,19,20]. The most frequent adverse reactions in the studied group were dermatological alterations in 14.27% of them. Compared to the study by Altcheh et al. (2011), these adverse reactions were much less frequent than in the 71% of the patients in the age range between 10 days and 19 years of age. The main adverse reactions to the specific drug in Argentina consisted of dermatological complaints, such as skin rash, eczema, pruritus, polymorphic erythema and urticaria (*16*). In the Brazilian series, clinical follow-up showed that 54.8% had positive serologies without signals of disease; 16.7% were considered cured for having negative sustained serologic conversion in serial evaluations and only 2.4% were considered to have mild chronic cardiopathy.

Finally, it was possible to identify that the initial clinical manifestation in children and adolescents varies from febrile syndrome to complex cases of acute myopericarditis. Among those who had cardiac involvement, severe acute myocarditis affected 18.8% of them with faster resolution after immediate specific treatment. When comparing the clinical manifestation of the disease in children and adults, it is evident that in both, febrile syndrome was the most frequent manifestation. Second, dyspnea and asthenia are frequent manifestations in adults, while pallor and headaches are predominant in children and youth. In children, therefore, the presumed diagnosis of chagasic etiology is mandatory in those with prolonged fever or acute myocarditis. Anemia was the most frequent nonspecific laboratory abnormalities in children and adolescents.

We are in debt with the affected people in search for diagnostic tools that could access the cure rates most faithful, especially in those exposed to vertical transmission. The decrease in antibodies IgG against *T. cruzi* titers during the mean follow-up period of 10.5 years, after treatment and maintenance of those in lower titers confers antiparasitic immune memory persistence, which could suggest low potential evolution to chronic phase of the disease, after treatment with drugs received during the acute phase.

## Figures and Tables

**Table 1:** Demographic and epidemiological data from patients with Chagas disease from Amazon region, Brazil.

Epidemiological Data	Frequency	%
Age (years)		
0–2	4	3.2
3–7	20	15.9
8–11	40	31.8
12–17	62	49.2
Gender		
Female	46	36.6
Male	80	63.4
Origin		
Pará State regions		
Baixo Amazonas	1	0.8
Marajó Island	26	20.8
Metropolitan area of Belém	52	41.2
Northeast	41	32.5
Southeast	2	1.6
Amapá State		
Santana	4	3.2
Transmission form		
Oral	86	68.3
Vectorial	19	15.1
Vertical	2	1.6
Undefined	19	15.1
Outbreak		
Yes	90	71.4
No	35	27.8
Undefined	1	0.8

**Table 2:** Main clinical manifestations in young patients with Chagas disease.

Clinical Manifestations	Frequency	%	Clinical Manifestations	Frequency	%
Fever	117	92.8	Hepatomegaly	34	26.9
Headache	74	58.7	Chills	27	21.4
Pallor	64	50.8	Caught	24	19.0
Myalgia	56	44.4	Splenomegaly	24	19.0
Abdominal pain	54	42.8	Exanthema	20	15.9
Edema of face	53	42.1	Edema of lower limbs	19	15.1
Adenopathy	49	38.9	Chest pain	13	10.3
Dyspnea	45	35.7	Palpitations	13	10.3
Arthralgia	44	34.9	Nodules in lower limbs	4	3.2
Diarrhea	34	26.9	Inoculation lesion	4	3.2

**Table 3:** Counts of blood cells according with age in young patients with Chagas disease before treatment.

Hematological Analysis	6–23 Months	2–6 y	7–9 y	≥10 y (Female)	≥10 y (Male)	Total N%	
Anemia	6	11	11	14	20	62	49.2
Reference hemoglobin concentration rate	2	6	6	3	9	25	19.8
Reference leukocytes count	3	10	15	11	27	66	52.3
Leukocytosis	5	6		2	1	14	11.1
Leukopenia		1	2	3	1	7	5.5
Reference platelets count	3	7	9	9	20	48	38.1
Thrombocytosis	5	8	4	4	7	28	22.2
Thrombocytopenia		2	1		1	4	3.2

**Table 4:** Electrocardiographic abnormalities of children patients with Chagas disease before treatment.

Electrocardiogram	N	%
Normal	52	59.1
Abnormal	36	40.9
Diffuse repolarization abnormalities	10	27.8
Sinusal tachycardia	6	16.7
Conduction disturbances	3	8.3
Sinusal arrhythmia	2	5.5
Incomplete right bundle branch block	2	5.5
Block in the superior division of the left branch	2	5.5
Bradycardia sinusal	1	2.8
Complete left bundle branch block	1	2.8
First degree atrio ventricular block	1	2.8

**Table 5:** Evolution observed in the end point post treatment of patients with Chagas disease.

Evolution after Treatment	Frequency	%
Sustained nonreactive serologic tests	21	16.7
Persistence of IgG antibodies against *T. cruzi* without evidence of cardiac disease	69	54.8
Persistence of IgG antibodies against *T. cruzi* with evidence of cardiac injury	3	2.4
Undefined	33	26.2
Total	126	100

## References

[1] Panamerican Health Organization (PAHO/WHO) (2009). Guide to Surveillance, Prevention, Control and Clinical Management of Acute Chagas Disease Transmitted by Food.

[2] Valente S.A.D.S., da Costa Valente V., das Neves Pinto A.Y., de Jesus Barbosa César M., dos Santos M.P., Miranda C.O.S., Cuervo P., Fernandes O. (2009). Analysis of an acute Chagas disease outbreak in the Brazilian Amazon: Human cases, triatomines, reservoir mammals and parasites. Trans. R. Soc. Trop. Med. Hyg..

[3] Pinto A.Y.D.N., Valente V.D.C., Coura J.R., Valente S.A.D.S., Junqueira A.C.V., Santos L.C., De Macedo R.C. (2013). Clinical Follow-Up of Responses to Treatment with Benznidazol in Amazon: A Cohort Study of Acute Chagas Disease. PLoS ONE.

[4] Pinto A.Y.N., Kimberlin D.W., Brady M.T., Jackson M.A., Long S.S. (2018). American Trypanosomiasis. Red Book:2018 Report of the Committee in Infectious Disease.

[5] Pinto A.Y.N., Valente S.A.S., Valente V.C., Ferreira A.R., Coura J.R. (2008). Acute phase of Chagas disease in the Amazon: Brazilian study of 233 cases from Pará, Amapá and Maranhão observed between 1988 and 2005. Rev. Soc. Bras. Med. Trop..

[6] Pinto A.Y.N., Valente S.A.S., Valente V.C. (2004). Emerging acute Chagas disease in Amazonian Brazil: Case reports with serious cardiac involvement. Braz. J. Infect. Dis..

[7] de Barros Moreira Beltrão H., de Paula Cerroni M., de Freitas D.R.C., das Neves Pinto A.Y., da Costa Valente V., Valente S.A., Costa E.D.G., Sobel J. (2009). Investigation of two outbreaks of suspected oral transmission of acute Chagas disease in the Amazon region, Pará State, Brazil, in 2007. Trop. Doct..

[8] Secretaria de Vigilância em Saúde (2019). Ministério da Saúde Brasil. Boletim Epidemiológico Doença de Chagas Aguda e Distribuição Espacial dos triatomíneos de Importância Epidemiológica, Brasil 2012 a 2016.

[9] Ostermayer A.L., Passos A.D.C., Silveira A.C., Ferreira A.W., Macedo W., Prata A.R. (2011). National seroprevalence survey evaluation of the control of Chagas disease in Brazil (2001–2008). Rev. Soc. Bras. Med. Trop..

[10] Dias J.C.P., Ramos N.A., Gontijo E.D., Luquetti A., Shikanani-Yasuda M.A., Coura J.R. (2016). II Consenso brasileiro em doença de Chagas, 2015. Epidemiologia e Serviços de Saúde.

[11] Rassi A., Ferreira H.O. (1971). Attempts of specific treatment of the acute stage of Chagas’ disease with nitrofurans in prolonged schemes. Rev. Soc. Bras. Med. Trop..

[12] Pinto A.Y.N., Farias J.R., Marçal A.S., Galucio S., Costi R.R., Valente V.C. Severe acute Chagas disease in the Amazon indigenous Brazilian. http://scielo.iec.gov.br/pdf/rpm/v21n2/v21n2a02.pdf.

[13] Shikanai-Yasuda M.A., Lopes M.H., Tolezano J.E., Umezawa E., Amato V.N., Barreto A.C., Higaki Y., Moreira A.A., Funayama G., Barone A.A. (1990). Acute Chagas’ disease: Transmission routes, clinical aspects and response to specific therapy in diagnosed cases in an urban center. Rev. Inst. Med. Trop..

[14] Alarcón de Noya B., Díaz-Bello Z., Colmenares C., Ruiz-Guevara R., Mauriello L., Zavala-Jaspe R., Suarez J.A., Abate T., Naranjo L., Paiva M. (2010). Large Urban Outbreak of Orally Acquired Acute Chagas Disease at a School in Caracas, Venezuela. J. Infect. Dis..

[15] Pinto A.Y.N., Ferreira S.M.A.G., Valente S.A.S., Valente V.C., Ferreira A.G. (2010). Electrocardiographic changes during and after treatment with benznidazole in acute stage of Chagas disease indigenous to the brazilian Amazon. Revista Pan-Amazônica de Saúde.

[16] Altcheh J., Moscatelli G., Moroni S., Garcia-Bournissen Freilij H. (2011). Adverse events after the use of benznidazole in infants and children with Chagas disease. Pediatrics.

[17] Meymandi S., Hernandez S., Park S., Sanchez D.R., Forsyth C. (2018). Treatment of Chagas Disease in the United States. Curr. Treat. Options Infect. Dis..

[18] Machado-de-Assis G.F., Diniz G.A., Montoya R.A., Dias J.C.P., Coura J.R., Machado-Coelho G.L.L., Albajar-Viñas P., Torres R.M., Lana M.D. (2013). A serological, parasitological and clinical evaluation of untreated Chagas disease patients and those treated with benznidazole before and thirteen years after intervention. Memórias do Instituto Oswaldo Cruz.

[19] Morillo C., Marin-Neto J.A., Avezum A., Sosa-Estani S., Rosas F., Villena E., Quiroz R., Bonilla R., Britto C., Guhl F. (2015). Benefit Investigators. Randomized trial of benznidazole for chronic Chagas’ cardiomyopathy. N. Engl. J. Med..

[20] Carlier Y., Torrico F., Russomando G., Luquetti A., Freilji H., Albajar Vinas P. (2011). Congenital Chagas disease: Recommendations for diagnosis, treatment and control of newborns, siblings and pregnant women. PLoS Negl. Trop. Dis..

